# A perception survey on the roles of nurses during triage in a selected public hospital in Kwazulu-Natal Province, South Africa

**DOI:** 10.11604/pamj.2020.37.9.22211

**Published:** 2020-09-02

**Authors:** Olunike Blessing Olofinbiyi, Makhosazane Dube, Euphemia Mbali Mhlongo

**Affiliations:** 1 School of Nursing and Public Health, College of Health Sciences, University of KwaZulu-Natal, South Africa

**Keywords:** Emergency care, KwaZulu-Natal, nurses’ perceptions, nursing roles, public hospital, triage

## Abstract

**Introduction:**

triage is gradually becoming an autonomous nursing role essential to patients' safety and the efficient delivery of emergency care. The increased need for more holistic and advanced care during triage makes the role of nurses during triage highly indispensable. However, several studies have shown that nurse-led triage has been so successful over the years in most African countries and in other developing countries. South African Triage Scale (SATS) is an example of triage tool that was designed in such a way that the lowest cadre nurse can successfully implement. The success recorded by this tool made most African countries and some other developing countries adopt the tool. The study was designed to explore the roles of nurses during triage in a selected public hospital in KwaZulu-Natal province.

**Methods:**

this study utilized a quantitative approach, in which a non-experimental survey involving convenience sampling technique was chosen as the most suitable sampling technique for the study. Recognition-primed decision model formed the framework of the study. Ethical clearance was obtained from University of KwaZulu-Natal Ethics Review Board and ethics principles were observed during the study.

**Results:**

the result of the study indicated that majority (100%) of the respondents perceived that nurses have lots of roles to perform during triage. They further unveiled that it is highly paramount for nurses to manage the waiting room and control overcrowding in the unit.

**Conclusion:**

the study draws on the need for qualified and experienced nurses to be in charge of these roles in order to reduce the mortality and morbidity rates that usually occur during triage administration.

## Introduction

An organization must have a stipulated set of roles for its staff in order to achieve its set goals and the term 'role' is a set of expectations about behaviour ascribed to a specific position in society [1,2]. In order to support this view, the role description should be designed in order to guarantee the best possible performance of employees [3]. Furthermore, triage is also an autonomous nursing role essential to patient safety and the efficient delivery of emergency care. In congruence with this view, the roles of nurses are highly indispensable during triage and several studies have shown that nurse-led triage has been so successful over the years in most African countries and in other developing countries [4]. South African Triage Scale (SATS) is an example of triage tool that was designed in such a way that the lowest cadre nurse can successfully implement [5]. The exceptional feature of SATS tool made SATS a friendly triage tool for most African countries and other developing countries [6,7]. The roles of nurses during triage are highly indispensable but most nurses are scared of these roles [8]. However, triage can either be front lined by nurses or physicians. The role of nurses during triage is critical, especially when nurses are at frontline [9]. Another study concurs that placing nurses at frontline during triage is indeed a critical and crucial role, especially on battle fields or war [10]. The roles of a nurse on the frontline are to approach scene with caution and upwind, carry out scene assessment, recognize signs and indicators of complex injuries, estimate the number of casualties/victims, estimate resource requirements, carry out primary triage, administration of medical care, and transport [11]. Conversely, before a nurse can perform triage, he or she must be filled with reservoir of knowledge, experience and must be able to think critically in order to make the right decision in an environment where available data source is limited and incomplete. Because the crucial and ultimate goals of a nurse fronting triage are to ensure preservation of life, caring for the victims and returning the greatest possible numbers of victims [12]. Furthermore, nurse-led triage had been found to reduce waiting time for patients in South Africa and in some other developing countries that have adopted this tool.

Another comparative study between the nurse practitioner and general practitioners demonstrated that the nurse practitioner can deal with patients effectively and they can be assumed as superb candidates for expanded and extended role [13]. Similarly, several studies have suggested the expansion of nurses´ role during triage in order to reduce waiting times for non-emergent cases [14,15]. Conversely, it was argued that physician-led triage is more reliable than nurse-led triage and some EDs have introduced physician-led triage with the aim of improving emergency care [16]. The most essential roles of a nurse in charge of triage are to ensure assessment of patients by observation of general appearance, collection of a focused history in order to identify the presenting problems and clinical risk [17]. This same study also unfolds that nurses must ensure the collection and interpretation of physiological data using a primary survey approach and quickly institute first aid measures where necessary. Similarly, the establishment of quick first aid measure usually prevents increased mortality rate. Furthermore, the first role of a nurse in charge of triage is to briefly assess the patients within five to ten minutes, in a case where there is no overcrowding, the nurse can reassess patients based on his or her discretion [18]. Conversely, the average length of time to triage a patient was five minutes but there is a significant increase in triage time when patients are triaged to a specialty, expected by a specialty, or were actively “seen and treated” in triage [19]. Another study shows that early prioritization of patients is another essential role of every nurse dealing with triage and a nurse can only make the right prioritization after he or she might have undergone some professional training in the triage system [20]. Congruently, the main role of a triage nurse is to first prioritize patients with life-threatening or emergency conditions and initiate appropriate interventions [21]. While, some studies argue that nurses should have wide variability in their degree of experience, preparation and orientation before they should be allowed to engage in the prioritization of patients´ needs in order to ensure adequate life safety [21,22]. The officers in charge of emergency units should also ensure that nurses must have worked in the emergency unit for like two to three years in order to gain experience with many different conditions before giving them the chance to triage patients [23].

The responsibility of the Nurse in charge of triage is to escalate and engage further assistance where appropriate. It is also the responsibility of the nurse to manage the patients on queue but this should be consistently done by a triage nurse [24]. However, another study suggests that the Emergency Departments (Eds) may choose to implement strategies to manage the queue according to local needs. While there was an emphasis on how important it is for a Nurse to be involved in identification and promotion of cultural safety for patients that are triaged. There is also a need for the nurse to access the interpreter service and appropriate information regarding triage in order to reduce the waiting time [24,25]. Nurses must assess patient subjectively and objectively, re-assess patients who are waiting, initiate emergency treatment if necessary, manage and communicate with patients in waiting room, provide education to patients and families when necessary, sort patients into priority groups according to guidelines, transport patients to appropriate treatment areas, communicate status of patients to other concerned health professionals and ensure proper documentation of all cares rendered by the nurse [25]. In line with this study, communication is one of the essential roles of nurses during triage. Therefore, nurses as the key members of the health care team, their roles are to establish communication with other healthcare professionals, patients and relatives in order to facilitate the process of care [26]. In addition, a junior nurse should be able to triage patients in the emergency unit, therefore releasing the registered nurse to provide more comprehensive care, such as urinalysis, ECGs and pregnancy tests [27].

Furthermore, a nurse needs to take note of some protective measures in order to prevent and control infection. This same study also unfolds that triage nurses need to double gloves, wear boots, gown and a self-contained breathing apparatus which may be used to prevent infection. While, a registered nurse may be required to initiate further advanced investigations such as X-rays and phlebotomy procedures in the developed country but in the low-income countries, a nurse must be able to front triage. This same study also reveals that there are some volunteers that do perform triage in some developing countries [27,28]. Some other roles of nurses during triage include evaluation of patients' vital signs, asking questions about their medical history, being clinically experienced, showing determinations about the urgency of a patient's need, having good judgment and leadership, being calm under stressful situations, having ability to make quick and right decisions, being knowledgeable about available resources, having a sense of humour, being a creative problem-solver, being available and well-experienced, being knowledgeable of anticipated emergency unit patients, having a high level of listening and communication skills as well as extensive knowledge of warning signs and symptoms [29]. In support of this view, good assessment skill and establishment of good judgment while making decisions are essential roles that every nurse performing triage must do [29,30].

Another study unfolds that a triage nurse must be able to make decisions based on scientific evidence. He or she must be committed to the patient’s bill of rights, must have empathy towards patients, must respect patients´ culture and value, must be able to manage ethical dilemmas and he or she must have good interpersonal skills. This same study also shows that a nurse in charge of triage must be the first person to assess patients, must ensure proper documentation of all rendered cares, must render nursing care holistically, must follow the organization´s guidelines during decision and be a good researcher [30]. In addition to the previous findings, communication between nurses and other health professionals, nurses and patients, nurses and relatives as well as among nurses themselves, is so essential during triage [31]. This same study also unfolds that most nurses do neglect this essential role; while ineffective communication, whether between patients and nurses, or between nurses themselves, remain a wide gap. Furthermore, communication within health sector can be in form of non-verbal (documentation) or verbal communication. It is the role of a professional triage nurse to provide efficient care, proper documentation and smoother communication between patients and healthcare providers [31,32]. This same study revealed further that failure to communicate and document properly always deteriorates the progress of triage in E.Ds. While, nurses must ensure proper documentation into triage flow sheet [32]. In addition to the previous findings, a triage nurse improves communication network, streamlines the reporting and workers´ compensation process, and increases satisfaction on all accounts. This same study discusses further that within the stipulated time, a triage nurse should send all necessary documentation (such as injury reports, injury details, and medical history) to the appropriate parties (including the medical provider, if medical service is recommended), and channel the injured to the company´s preferred providers [33].

In order to secure patient´s safety in triage room, the Emergency Nursing Associations (ENA) developed a position statement indicating the qualifications needed to be acquired by nurses before they can perform triage [34]. Congruently, a nurse must have undergone some educational trainings on triage and acquire some set of qualifications on triage before he or she should be allowed to perform triage. Furthermore, Emergency Nurses Association (ENA) indicated several educational programs that a nurse should be enrolled for before he or she can perform triage. This association enumerates some programs such as Cardio-Pulmonary Resuscitation (CPR), Advanced Life Support (ALS), Emergency Nursing Paediatric Course (ENPC), Trauma Nursing Core Course (TNCC), Geriatric Emergency Nursing Education (GENE), Certified Emergency Nurse (CEN), and Certified Paediatric Emergency Nurse (CPEN) [34,35]. Conversely, sorting of patients should not be based on educational qualifications alone but also need to acquire some set of interpersonal skills such as, interdisciplinary skill, critical thinking skill, communication skills and decision-making skills [36]. However, competency of nurses is highly essential before they can perform triage. Because, competency serves as a level of performance demonstrating the effective application of knowledge, skill and judgement. In congruence with this notion, a statement released by the international council of Nurses confirmed that competency is the ability to perform according to predefined standards. Competency can only be gained through work experience, education, mentors and training. The current study is a systematic approach to discern the roles of nurses during triage, attempting to uncover the interface between patients and hospital services - a clinical approach that successfully links the role of nurses to patients´ satisfaction during triage administration [37].

## Methods

The study was conducted at one of the public hospitals in the capital of KwaZulu-Natal province, South Africa. The population comprised the nurses in the Emergency Department (ED), Paediatric Unit (PU) and Outpatient Department (OD) at the selected public hospital, Grey´s hospital. This study recruited nurses working in ED, PU and OPD because these are the major units where triage is performed. These three departments were chosen so that a cross sectional idea of the perceptions of the various units performing triage could be explored.

**Respondents:** the researcher used a sample size of 100 respondents to obtain data information for the study. A non-probability, convenience sampling method was used to recruit 24 nurses in emergency department, 38 nurses in Paediatric unit and 38 nurses in outpatient department. Furthermore, the researcher chose the elements of the study that were available and ready at the right place and at the right time during the study period. In this study, convenient sampling was the most suitable technique because it made it easier for the researcher to collect data from the participants, especially when the nurses were off duty. Data collection was carried out between 01 August and 21 September 2018, with a response rate of 100%. The researcher collected the data personally with other two research assistants at the selected institution after obtaining permission.

**Analysis:** data were generated, organized and analysed using the Statistical Package for the Social Sciences (SPSS) Version 25.0. Descriptive statistics was used to determine the mean, mode and the overall perception of the respondents. The primary instrument used for the data collection was a questionnaire.

**Ethical considerations:** nursing research must not only have the potential to generate and refine knowledge, but must be ethical in its development and implementation. Ethical approval was granted to conduct the research from the University of KwaZulu-Natal´s Biomedical Research Ethics Committee. The protocol reference number is HSS/0547/018M. Before the full approval of the ethical clearance was granted, the Department of Health Research unit approved the research proposal and the HRKM reference number is 224/18, while the NHRD reference number is KZ_201806_008. Permission was also granted from the selected hospital where this study was conducted. A two-page participation information document was provided to each person explaining the purpose of the research and the nature of the questionnaire. They were also provided with consent form to participate in the study which they signed before answering any questions. Each of the respondents was given a copy of the consent form. The principle of justice was adhered to by ensuring the respondents´ confidentiality. During data collection processes, the researcher informed the respondents not to write their names on the questionnaires. The respondents were made to understand that participation was strictly voluntary. They have the right to renegotiate consent during the research process or to ignore particular questions without giving an explanation and they can withdraw from the study at any point. The respondents were also encouraged to ask any questions they wished in relation to the study and they were provided with written consent forms to be signed and give back to the researcher. All prospective participants were informed of the purpose of the study and of the fact that the research results would be made available to all respondents. The respondents had the right to decide voluntarily whether or not to participate in the study without any risk of penalty or prejudicial treatment. The principle of respect was thus adhered to.

## Results

A total number of 100 questionnaires were administered to the respondents of which the entire 100 questionnaires were returned, giving a 100% responses rate. Majority (96%) of the respondents were females, while 4% (n=4) were males. The study also showed that 37% (37) of the respondents were between the ages of 41-50, while 30% of the respondents were between the ages of 31-40years. Only 7 (7%) were between the ages of 20-30. Forty nine (49%) of the respondents agreed that it is the role of nurses to frontline triage. They further unveiled that it is part of nurses´ roles to initiate contact with patients and family. Similarly, over half (52 %) of the respondents agreed with the role of keeping patients and relatives informed of delays and expected time to be seen. It was also shown in this study that majority 55% (n=55) of the respondents agreed that it is the role of nurses to institute first aid measures where necessary, while 39% (n=39) of the respondents strongly agreed that it is part of their roles to institute first aid measures where necessary, this same table below also demonstrates the findings on assessment (history taking) of patients and it revealed that 61% (n=61) of the respondents agreed with this role, while 29% (n=29) of the respondents strongly agreed.

Findings on taking of adequate physical examination and vital signs showed that majority (56%) of the respondents perceived that the nurses should ensure the performance of adequate physical examination and taking of the patients vital signs, 37% (n=37) of the respondents strongly agreed. The view of the respondents on the role of nurses performing brief investigations such as urinalysis and PCV (Packed Cell Volume) shows that most of the respondents, about 68% (n=68) agreed with this role, while 23% (n=23) strongly agreed. The findings on prioritization of patients showed that majority 51% (n=51) of the respondents agreed with the performance of this role, while 45% (n=45) of the respondents strongly agreed with this role, while none of the respondents disagreed with this role of adequate physical examination and taking of the patients´ vital signs. It was also unveiled that documentation is highly essential when nurses are carrying out their roles during triage, because 54% (n=54) and 41% (n=41) of the respondents agreed and strongly agreed with this role respectively, while the mean was 4.36. Out of 100 respondents, majority of the respondents, about 56% (n=56) agreed that it is the role of nurses to control infection, while 38% (n=38) of the respondents strongly agreed and the mode of the findings was 4 (agreed) while the mean was 4.32. Lastly, findings displayed on the table below show that 57% (n=57) of the respondents agreed that, it is part of nurses´ role to manage patient flow of triage through the unit while 39% (n=39) strongly agreed with the role (Table 1) The findings of the cross tabulation between the respondents´ years of work experience and the roles of nurses during triage unveiled that there is a significant relationship between the nurses´ years of work experience and the nurses´ roles during triage, given the values of the findings as: p-value of 0.001, df: 32 and the Chi-square was 101.481. These findings revealed that the years of the nurses´ work experience also determine the type of roles they can perform during triage (Table 2).

**Table 1:** percentage distribution of respondents on the roles of nurses during triage

Roles of Nurses	Strongly disagree	Disagree	Neutral	Agree	Strongly agree	Total (%)
The nurse must be at the frontline of the unit	0% ( n=0)	1% (n=1)	6% (n=6)	49 (n=49)	44 (n=44)	100% (n=100)
Manage the waiting room and control overcrowding.	0% (n=0)	0% (n=0)	13% (n=13)	57% (n=57	30% (n=30)	100% (n=100)
Initiate contact with patients and family	1% (n=1)	2% (n=2)	9% (n=9)	53 (n=53)	35 (n=35)	100% (n=100)
Keep patients and relatives informed of delays and expected time to be seen	0% (n=0)	1% (n=1)	9% (n=9)	52% (n=52)	38% (38)	100% (n=100)
Institute first aid measures where necessary	0% (n=0)	1% (n=1)	5% (n=5)	55% (n=55)	39% (n=39)	100% (n=100)
Assessment (history taking) of patients	0% (n=0)	0% (n=0)	10% (n=10)	61% (n=61)	29% (n=29)	100% (n=100)
Focused physical examination and taking of vital signs	1% (n=1)	0% (n=0)	6% (n=6)	56% (n=56)	37% (n=37)	100% (n=100)
Brief investigations such as urinalysis and PCV	1% (n=1)	2% (n=2)	6% (n=6)	68% (n=68)	23% (n=23)	100% (n=100)
Prioritization of patients	0% (n=0)	0% (n=0)	4% (n=4)	51% (n=51)	45% (n=45)	100% (n=100)
Documentation	0% (n=0)	0% (n=0)	5% (n=5)	54% (n=54)	41% (n=41)	100% (n=100)
Infection control	0% (n=0)	0% (n=0)	6% (n=6)	56% (n=56)	38% (n=38)	100% (n=100)
Manage patient flow through the unit	0% (n=0)	0% (n=0)	4% (n=4)	57% (n=57)	39% (n=39)	100% (n=100)

**Table 2:** cross-tabulation of nurses' years of work experience and their roles during triage

Years of work experience/roles	p-value	Df	Chi-Square
	0.001	32	50.799
Total number of Valid Cases	100		

**Limitations to this study:** the study was limited to only one hospital in KwaZulu-Natal province and the study only made use of non-probability convenience sampling, so the findings cannot be generalized to all the provinces in South Africa. The research adopted a quantitative methodological approach as the only method of data collection. While it is believed that qualitative method could have generated more detailed information about the subject of the research.

## Discussion

The findings of this study showed that, 49% and 44% of the respondents agreed and strongly agreed respectively that it is the role of nurses to frontline triage. This finding revealed that majority of the respondents perceived that it is the role of nurses to frontline triage. This finding is in consonance with a study that establish front lining triage as the first role of a nurse but there is need for an experienced professional nurse to front triage in order to improve patients´ flow and make right decisions. In addition, the roles of a nurse on the frontline are to approach scene with caution and upwind, carry out scene assessment, recognize signs and indicators of complex injuries, estimate the number of casualties/victims, estimate resource requirements, carry out primary triage, administration of medical care, and transport [38]. The findings on the management of waiting room and control of overcrowding revealed that most (57%) of the respondents agreed that the management of waiting room and control of overcrowding is part of nurses´ roles during triage, while 13% of the respondents strongly agreed. Another similar findings in the study unveiled that 57% of the respondents agreed that, it is part of nurses´ role to manage patient flow of triage through the unit, while 39% strongly agreed with the role. In support of the above mentioned findings, nurses need to accelerate the delivery of patients with life-threatening conditions, aid categorization of patients according to their clinical manifestation and decrease overcrowding. Also, the management of overcrowding can only be fully achieved by an effective collaboration of the whole health professionals [39].

The findings from this study revealed that 53% of the respondents agreed and 35% of the respondents strongly agreed respectively that it is part of nurses´ roles to initiate contact with patients and family. Another similar finding in this study unveiled that just over half (52) of the respondents agreed with the role of keeping patients and relatives informed of delays and expected time to be seen, while 38% strongly agreed that it is the role of nurses to keep patients and relatives informed of any delays and expected time to be seen. It is part of nurses´ roles to inform the patients on what to do, what is happening and what next to do including procedures and treatments as nurses are the main advocate between patient and other health professionals [40]. Findings on assessment (history taking) of patients revealed that 61% of the respondents agreed with this role, while 29% of the respondents strongly agreed with this role and out of 100% respondents, majority (56%) of the respondents perceived that the nurses should ensure the performance of adequate physical examination and taking of the patients vital signs, 37% of the respondents strongly agreed. These findings showed that despite how rapid triage needed to be implemented, physical assessment of patients is highly essential. These findings are in conformity with one study that states “the essential roles of a nurse in charge of triage are to ensure assessment of patients by observation of general appearance, collection of a focused history in order to identify the presenting problems and clinical risk”. In addition, nurses must also assess patient subjectively and objectively [41]. It was also shown in this study that majority (55%) of the respondents agreed that it is the role of nurses to institute first aid measures where necessary, while 39% of the respondents strongly agreed that it is part of nurses´ role to institute first aid measures where necessary. Another study in line with this finding unfolds that nurses must ensure the collection and interpretation of physiological data using a primary survey approach and quickly institute first aid measures where necessary [41,42].

The findings on prioritization of patients show that majority (51%) of the respondents agreed with the performance of this role, while 45% of the respondents strongly agreed with this role. Another study in line with this finding unveiled that early prioritization of patients is another essential role of every nurse dealing with triage; nurses can only make the right prioritization after he or she might have undergone some professional training in the triage system. Furthermore, the main role of a nurse during triage is to first prioritize patients with life-threatening or emergency conditions and initiate appropriate interventions [42]. This study also state that documentation is highly essential when nurses are carrying out their roles during triage, because 54% and 41% of the respondents agreed and strongly agreed with this role. A nurse in charge of triage must be the first person to assess patients and he or she needs to ensure proper documentation of all rendered cares. While, the role of a professional triage nurse is to provide efficient care, proper documentation and smoother communication between patients and healthcare providers [43]. The result from the current study shows that out of 100 respondents sampled for the study, majority (56%) of the respondents agreed that it is the role of nurses to control infection, while 38% of the respondents strongly agreed. These nurses perceived that, despite how rapid triage needed to be implemented, nurses need to prevent and control infections perfectly. Congruently, there is need for a nurse to wear appropriate protective measures in order to prevent and control infection during triage. This same study also unfolds that triage nurses need to wear double gloves, wear boots, gown and a self-contained breathing apparatus in order to prevent infection [43,44]. The overall findings of the respondents on nurses roles during triage indicated that none of the respondents had negative perception towards the roles of nurses during triage, while 100% (n=100) had positive perception towards the roles of nurses during triage. In congruence with this finding, the roles of nurses are highly indispensable during triage; and several studies have shown that nurse-led triage has been so successful over the years in most African countries and in other developing countries [44].

## Conclusion

The study draws on the need for qualified and experienced nurses to be in charge of the triage roles in order to reduce mortality and morbidity rates that usually occur during triage administration. The outcome also revealed that most of the respondents were females, while the average age of the respondents were between 41-50 years. It emerged from this study that majority of the nurses were filled with reservoir of experience on triage. Through the study, it was unfolded that it is essential for nurses to be filled with experience and skills before performing or fronting triage. However, some of the respondents selected for the study were of the opinion that nurses, especially new recruits, need to undergo some specific training before performing or fronting triage. Based on the findings of the study, larger proportion of the nurses perceived that the role of nurses during triage is tremendously indispensable, as most of the respondents perceived that nurses have a lot of roles to perform during triage administration.

**Policy recommendations:** according to the study, triage has been a very crucial tool that allows nurses to think critically in order to reduce mortality and morbidity rates. In order to meet the patients´ needs more efficiently, there is a crucial need for nurses to update their knowledge, which can adequately be acquired through education. Based on the findings and the literatures reviewed for the study, the researcher recommends the following: 1) In order to reduce mortality rate and provide perfect care, there is need for a qualified and an experienced nurse to front triage. 2) The hospital management should provide more experienced and professional nurses in order to aid the flow of triage. 3) There is need for improvement on the proper documentations during triage. 4) All nurses should be granted equal opportunities by the hospital management to attend formal courses on triage. 5) There is need for Nursing Education Institutions (NEI) to make triage one of the core courses to be taken at nursing colleges, so as to familiarize student nurses with triage tool. 6) The policy makers should approve and endorse some laboratory tests that nurses can quickly do, especially during mass casualty.

**Recommendations for further studies:** 1) This study is a context-specific research that was conducted at one hospital of KwaZulu-Natal province. A replicate study involving all provinces of the country should be conducted in order to garner broader perspective which could be generalized across the nation and globally. 2) Upcoming researchers should make use of qualitative or mixed method in order to create opportunities for comparative research findings in terms of future methodological approach.

### What is known about this topic

- Triage is gradually becoming an indispensable tool globally;
- African countries suffer the highest rates of every category of injury;
- The South African Triage Scale (SATS) is a nurse-led and in-hospital triage tool.

### What this study adds

- Only nurses with formal educational training, experience and skills should be at the frontline of triage administration;
- Management of waiting room and control of overcrowding is a significant role of nurses during triage;
- Control of infections while sorting patients is an essential role of nurses to avoid nosocomial infections.
